# Supplementary figures and images for: An Epilepsy-Associated CILK1 Variant Compromises KATNIP Regulation and Impairs Primary Cilia and Hedgehog Signaling
Source: Cells. 2024 Jul 26;13(15):1258. doi: 10.3390/cells13151258 (PMC11311665; doi:10.3390/cells13151258)

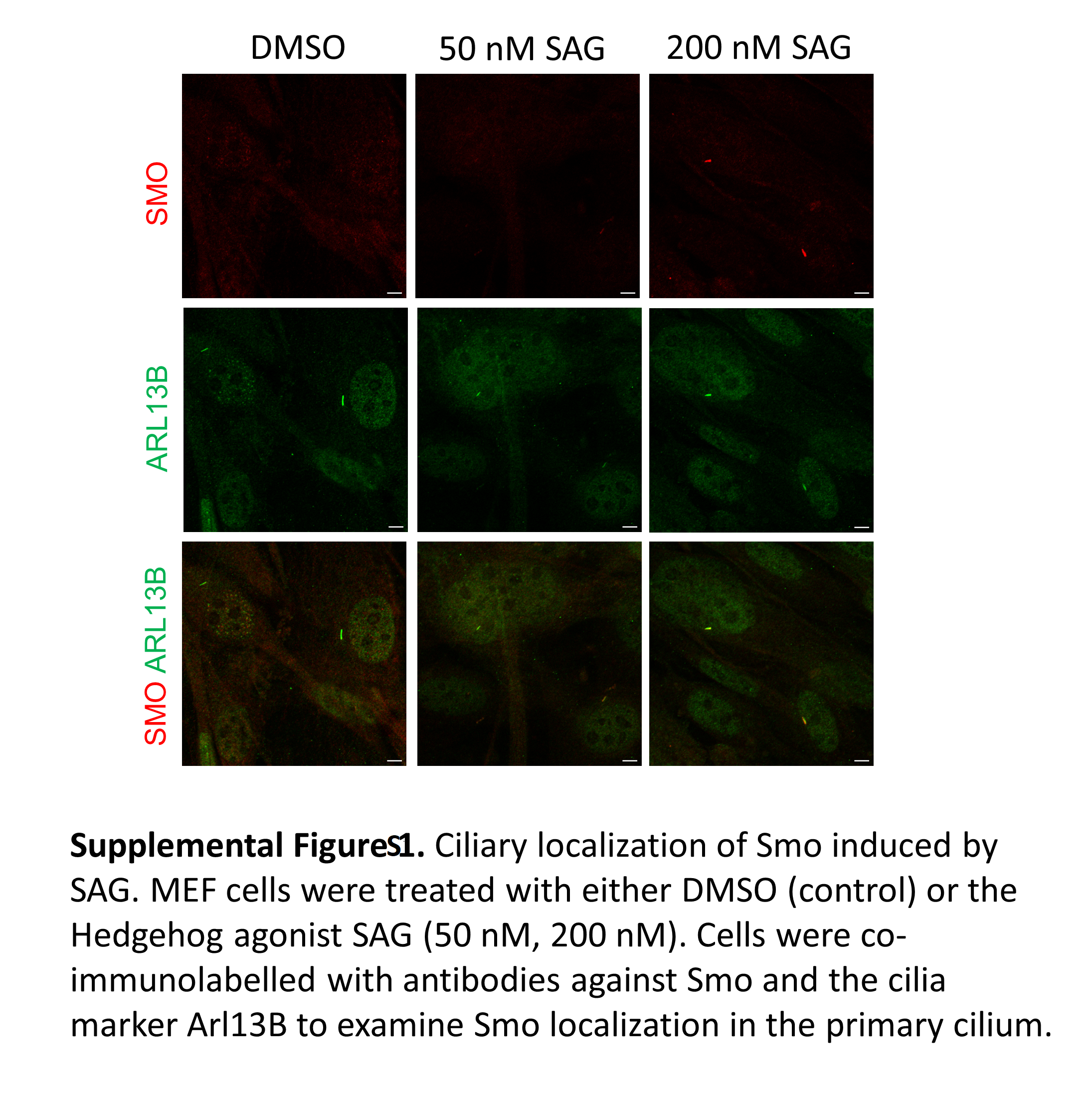

Supplement: Supplementary file 1 [file cells-13-01258-s001.zip › Figure S1.tif]
